# Genetic Factors Associated with Suicidal Behaviors and Alcohol Use Disorders in an American Indian Population

**DOI:** 10.21203/rs.3.rs-2950284/v1

**Published:** 2023-05-31

**Authors:** David Gilder, Rebecca Bernert, Katherine Karriker-Jaffe, Cindy Ehlers, Qian Peng

**Affiliations:** Stanford University; The Scripps Research Institute; The Scripps Research Institute

## Abstract

American Indians (AI) demonstrate the highest rates of both suicidal behaviors (SB) and alcohol use disorders (AUD) among all ethnic groups in the US. Rates of suicide and AUD vary substantially between tribal groups and across different geographical regions, underscoring a need to delineate more specific risk and resilience factors. Using data from over 740 AI living within eight contiguous reservations, we assessed genetic risk factors for SB by investigating: (1) possible genetic overlap with AUD, and (2) impacts of rare and low frequency genomic variants. Suicidal behaviors included lifetime history of suicidal thoughts and acts, including verified suicide deaths, scored using a ranking variable for the SB phenotype (range 0–4). We identified five loci significantly associated with SB and AUD, two of which are intergenic and three intronic on genes *AACSP1*, *ANK1*, and *FBXO11*. Nonsynonymous rare mutations in four genes including *SERPINF1* (PEDF), *ZNF30*, *CD34*, and *SLC5A9*, and non-intronic rare mutations in genes *OPRD1*, *HSD17B3* and one lincRNA were significantly associated with SB. One identified pathway related to hypoxia-inducible factor (HIF) regulation, whose 83 nonsynonymous rare variants on 10 genes were significantly linked to SB as well. Four additional genes, and two pathways related to vasopressin-regulated water metabolism and cellular hexose transport, also were strongly associated with SB. This study represents the first investigation of genetic factors for SB in an American Indian population that has high risk for suicide. Our study suggests that bivariate association analysis between comorbid disorders can increase statistical power; and rare variant analysis in a high-risk population enabled by whole-genome sequencing has the potential to identify novel genetic factors. Although such findings may be population specific, rare functional mutations relating to PEDF and HIF regulation align with past reports and suggest a biological mechanism for suicide risk and a potential therapeutic target for intervention.

## 1. INTRODUCTION

Suicide is a preventable public health problem that ranks as the second leading cause of death among young adults residing in the US (1). Suicide rates remain even higher among American Indians/Alaska Natives (AI/AN), with rates more than 50% greater when compared to the general U.S. population across all age groups (2–5). While suicide rates are unquestionably higher in the AI/AN population as a whole as compared to other US populations, rates also vary based on geographic region, tribal affiliation and whether one is living on a reservation (6–12).

American Indian/Alaska Natives also suffer from a disproportionate burden of the effects of alcohol, tobacco, and drug dependence (13, 14). Large-scale U.S. epidemiological studies indicate that, compared with other U.S. ethnic groups, AI /AN demonstrate the highest rates of alcohol and other drug dependence (15, 16). Additionally, lifetime rates of alcohol use disorders (AUD) differ depending on the tribes evaluated. Among individual tribal groups studied, reported rates of AUD have ranged from 20–70% (17–20)—significantly higher than epidemiological rates of AUD (14%) in the U.S. general population (21).

Substantial comorbidity has been demonstrated between SB and AUD. Chronic alcohol use is a clear risk factor for suicide (22), and having a severe use disorder and experiencing substance associated depression is particularly linked to increased risk for suicide (23). Being acutely intoxicated provides additional risk for lethal suicide over and above the risk of chronic use (24, 25). This is particularly true among American Indian/Alaska Natives (25, 26). However, documenting comorbidity between disorders does not necessarily imply a causal connection between them or a common etiological pathway. Both AUD and suicide have been shown to have a significant genetic component to their etiology (27, 28). Behavioral genetics studies have the advantage of being one of the strongest methods for determining whether the comorbidity among psychopathological conditions may be due to shared etiologies and/or pathologies associated with the disorders. For instance, a new study recently estimated the genetic correlation between suicide attempt and alcohol dependence at 44% in populations of European ancestry (29).

In recent years, numerous large genome-wide association studies (GWAS) have been reported for suicidal behaviors (28, 30–34). These studies were carried out in populations dominated by individuals of European descent. Genetic epidemiology studies have estimated the heritability of SB ranging from 17–55% (35, 36). Single nucleotide polymorphism (SNP)-heritability of suicide attempt using common genomic variants across the genome has been estimated to range from 3.5% in the UKBiobank (37) to 6.8% in the International Suicide Genetics Consortium (ISGC) (32). The largest GWAS meta-analysis to date (with ~ 959K individuals) reported 12 loci for suicide attempt. Risk loci were mostly intergenic and implicated genes *DRD2*, *SLC6A9*, *FURIN*, *NLGN1*, *SOX5*, *PDE4B*, and *CACNG2* (30). Intriguingly, one of the genes, *ROBO2*, reported by the ISGC GWAS to be associated with SB (31) is on a rare run-of-homozygosity (ROH) segment that was previously reported to be linked to severe AUD in an American Indian population (38).

While SNP-heritability and GWAS studies primarily estimate the impacts of common genomic variants, recent studies have shown that rare variants, especially those in low linkage disequilibrium (LD) with neighboring variants, are enriched for heritability for complex traits and diseases. Rare variants often represent recent and potentially deleterious mutations that can have biological consequences. Studies have indeed shown that most rare missense alleles in humans are deleterious (39). In fact, a recent Utah Suicide Genetic Risk Study (USGRS) interrogating rare protein-coding variants included on the Illumina PsychArray chip has identified five rare variants for suicide death (40). Genotyping chips, however, are usually not designed to capture rare variants in a population. Thus, to facilitate comprehensive studies of the impact of rare variants on a disease, exome sequencing or whole-genome sequencing is usually necessary.

The present report is part of a larger study exploring risk factors for substance dependence and suicide behaviors among American Indians (19, 41–43). This American Indian (AI) population has been sequenced and deep phenotyped. The lifetime prevalence of AUD and suicide in this AI population is high, and evidence for heritability, linkage to specific chromosome locations, and genome-wide findings for AUD have been demonstrated (38, 42, 44–47). The present study aimed to evaluate genetic factors associated with SB in this AI population. We hypothesized that certain genetic variants may underlie both SB and AUD, and further, that joint association analysis of related traits may lead to increased power to detect variants that contribute to both phenotypes. To this end, we conducted a bivariate genome association analysis between SB and AUD. We further hypothesized that rare variants in a high-risk population maybe a significant component of the complex genetic architecture underlying the disorder. We thus conducted gene-based and pathway-based rare and low-frequency variant analyses for SB.

## 2. MATERIAL AND METHOD

### 2.1 Participants

American Indian participants were recruited from eight geographically contiguous reservations with a total population of about 3,000 individuals. To be included in the study, participants had to be between the ages of 18 and 70 years, and mobile enough to be transported from their home to The Scripps Research Institute (TSRI). More details are given in Supplemental Materials. The protocol for the study was approved by the Institutional Review Board (IRB) of TSRI, and the board of the Indian Health Council, a tribal review group overseeing health issues for the reservations where the recruitment was undertaken. Written informed consent was obtained from each participant after the study was fully explained.

### 2.2 Phenotypes and genotypes

Potential participants first met individually with research staff, and during a screening period, participants completed a questionnaire that was used to gather information on demographics, personal medical history, and drinking history (48). Each participant also completed an interview based on the Semi-Structured Assessment for the Genetics of Alcoholism (SSAGA) (49), which was used to collect lifetime history of two types of self-directed violence: suicidal thoughts including ideation (Have you ever thought about killing yourself?) and/or plans (Did you have a plan? Did you actually consider a way to take your life? What were you going to do?), as well as suicidal acts, including suicide attempt history (Have you ever tried to kill yourself? How did you try to kill yourself?) reported by the participants and suicide deaths obtained from community sources (e.g., verified by public records, family/tribal informants) over an 8 year period. From the lifetime history of suicidal thoughts and acts, we defined the suicidal behavior phenotype as a ranking variable: 0-none, 1-ideation, 2-plans, 3-attempts, 4-death.

Diagnoses of lifetime DSM-5 AUD (mild, moderate, or severe) were also generated using the SSAGA. In addition, the interview retrospectively asks about the occurrence of alcohol-related life events, and the age at which the problem first occurred, from which a quantitative phenotype, the severity level of AUD, was derived. Briefly, it is indexed by 36 alcohol-related life events (Table S1) in the clinical course of the disorder (19, 50), with life events given a severity weight of 1 for events 1–12; 2 for 13–24; and 3 for 25–36. AUD severity was then calculated as the sum of the severity weights of the 36 life events (46). The relation between the SB phenotype and the AUD severity in this AI population is illustrated in Supplemental Figure S1. The Pearson’s correlation between SB and AUD severity is 0.3, with a 95% confidence interval of 0.23–0.36.

Participants had low-coverage whole genome sequencing on blood-derived DNA using a previously described pipeline (51). Further details can be found in the Supplementary Method.

### 2.3 Bivariate association analysis for suicidal behaviors and alcohol use disorders (SB-AUD)

We conducted genome-wide bivariate association analysis to identify genetic variants that might be associated with both SB and AUD severity (denoted as SB-AUD). By leveraging cross-trait covariance, multivariate tests for association may provide increased power over univariate tests. This occurs when the residual correlation between traits is opposite in direction to the genetic correlation induced by the genetic loci (52). To control for population admixture and familial relatedness, we used the multivariate linear mixed model implemented as *genome-wide efficient mixed-model association* (GEMMA) for the study (53). The bivariate association for each variant was conditioned on a genetic relationship matrix of the cohort derived from the genotypes, thus capturing a wide range of sample structures. Gender, age, and age-squared were further included as covariates. Variants with minor allele frequency (MAF) lower than 1% were excluded from this analysis.

### 2.4 Gene-based rare and low-frequency variant analysis for suicidal behaviors

We included both rare (MAF < 1%) and low-frequency (1% <= MAF < 5%) variants in a second set of analyses. We use the term “rare variants” broadly in this text to refer to variants with MAF < 5%. Rare variants are usually tested by aggregating them into groups. We analyzed the rare and low-frequency variants across the genome using SKAT-O that optimally combines a burden test and a non-burden sequence kernel association test (SKAT) (54) within a linear mixed model as implemented in EPACTS (55). The variants were grouped by genes or by pathways and tested against the SB phenotype. Gender, age, and age-squared were included as covariates in all sets of analyses.

For each gene, we formed two types of groups. One group considered all variants on exons, 5’ and 3’ untranslated regions (UTRs), upstream and downstream of the gene (denoted as *ExonReg*). The other group included only the nonsynonymous variants and the splicing-site variants of the gene (denoted as *Nonsyn*). Intergenic and intronic variants were excluded in the present study. For each group type, a gene was excluded if fewer than three markers were found, or if less than 0.5% of the samples had any such markers on the gene. This resulted in 28,718 genes in the *ExonReg* group and 12,588 genes in the *Nonsyn* group. Association analysis was performed between each gene-based set and SB. False discovery rates (FDR) controlled by the Benjamini–Hochberg procedure (Benjamini and Hochberg, 1995) were used to set significant *p* values from the test statistics of the association tests. We combined two gene variant groups together for the multiple testing correction and report the FDR-adjusted *p* values (Yekutieli and Benjamini, 1999). Note that the correction is conservative since the *Nonsyn* variants on a gene are a subset of the *ExonReg* variants, and thus they are correlated.

### 2.5 Pathway-based rare and low-frequency variant analysis for suicidal behaviors

We utilized a collection of canonical pathways from the Molecular Signatures Database (MSigDB) version 7.5.1 (56, 57). There are total of 2,981 pathway gene sets in this release. Since a pathway may contain a large number of genes, to limit the number of variants included in each set for test, we only considered rare and low-frequency nonsynonymous and splicing-site variants (*Nonsyn*) on the genes within each pathway. The same lters as in the gene-based tests were applied. We performed SKAT-O tests on the *Nonsyn* variant group for each pathway to be associated with SB. FDR-adjusted *p* values are reported.

### 2.6 Functional Analysis

For the top variants identified in the bivariate association analysis and for the top genes identified in the rare variant analysis, we obtained the combined annotation dependent depletion (CADD v1.6) scores (58) for the variants included in the test to assess the deleteriousness of these variants. CADD integrates multiple functional annotations and genome-wide variant effect prediction models to produce a scaled C-score. We report the number of variants tested in each gene that have C-scores in the range of 15–20 (1–3% most deleterious mutations), 20–30 (0.1–1%), and over 30 (< 0.1%). We chose 15 as the lowest C-score to report as it happened to be the median value for all possible canonical splice site changes and nonsynonymous variants in CADD v1.0 (59).

The variants with *p*-value < 10^− 6^ from the bivariate analysis were annotated with genes, and the associated set of genes was then subjected to functional enrichment analysis using GENE2FUNC in FUMA version 1.5.1 (60). An integrated network analysis was completed using GeneMANIA (61). The same set of functional analysis was applied to the top genes from the rare variants analysis as well.

More details can be found in Supplemental Method.

## 3. RESULTS

A total of 743 participants had sequencing data and a non-missing phenotype for SB (*none [0]*, *suicidal ideation* [1], *suicide planning* [2], *suicide attempt* [3], *suicide death* [4]), and 742 participants had data on AUD severity (range 0–69, mean = 22.6, sd = 18.8).

### 3.1 Bivariate genome-wide significant variants associated with SB-AUD in AI

**Figure 1:**
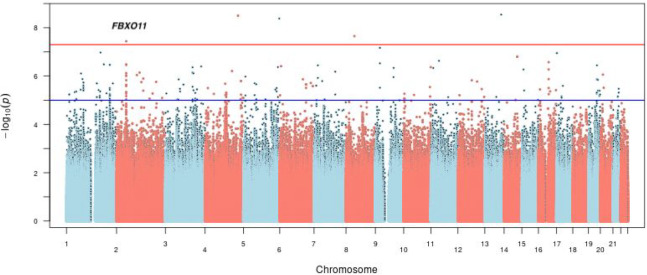
Manhattan plot of bivariate genome-wide association analysis for AUD severity and suicidal behaviors in AI.

Five variants were identified as being significantly associated with AUD severity and SB (*p* < 5E-8), as illustrated in Fig. 1. Only one of the variants, rs184204326 on gene *FBXO11*, has MAF over 5% (Table 1). Although the association of this variant is mostly driven by SB (*p* = 1.48E-07), the bivariate association is statistically more significant (*p* = 3.63E-08) than the univariate associations, which also holds for three other top variants, including an intergenic variant between genes *ZIC2* and *PCCA*, variant rs76300969 on gene *AACSL*, and variant rs530542541 on *ANK1*. Among the top variants shown in Table 1, rs184204326 is the only SNP for which brain expression quantitative trait loci (eQTL) were found in the Braineac database (62). That variant is associated with the differential gene expression of several genes in brain tissues including: putamen, substantia nigra, thalamus, occipital cortex, and temporal cortex (Table S2). The most significant eQTL is for *EPCAM* (encoding epithelial cell adhesion molecule) expression in the putamen tissue (*p* = 0.0019). The variant is also associated with gene expression in several brain tissues for *FBXO11*, *MCFD2* (encoding a soluble luminal protein), and *KCNK12* (encoding a potassium channel protein).

**Figure 2:**
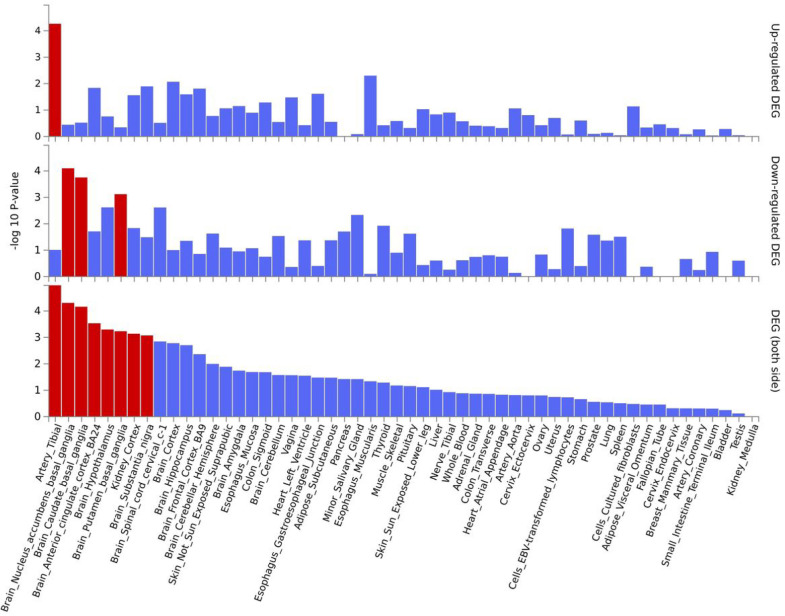
Enrichment in tissue-specific differentially expressed gene sets (DEG) of top genes associated with SB-AUD. The tissue-specific differential gene expression test was conducted against all genes across genomes that exhibited significantly increased or decreased expression levels in a certain tissue sample compared to all other samples. The analysis was performed using FUMA and utilized tissue-specific transcriptome data across 54 tissue types from GTEx v8. From top to bottom: up-regulated, down-regulated, differentially expressed. Red: Significantly enriched (*p* < 0.05 with Bonferroni correction).

Variants associated with SB-AUD at *p* < 10^− 6^ were mapped to 31 genes. The majority of these variants are intronic, upstream, or downstream from a gene. Rs1804145 on *SERPINF1* is the only nonsynonymous variant and has a C-score of 22.8 (top 0.52% most deleterious). These genes are enriched for 27 transcription factor (TF) targets and three microRNA targets (Table S3), suggesting that the top genes share certain regulatory motifs. These genes are most significantly up-regulated in artery, and down-regulated in areas included in the basal ganglia (nucleus accumbens, caudate, and putamen). In addition, they are significantly differentially expressed in anterior cingulate cortex, hypothalamus, substantia nigra, and kidney (Fig. 2). Many psychiatric or neurological disorders have been linked to basal ganglia targets including addiction and depression (63).

### 3.2 Genes with rare and low-frequency variants associated with suicidal behaviors in AI

Rare variant analysis identified six genes and one long intergenic non-coding RNA (lincRNA) as being significantly associated with SB (FDR < 0.05), and an additional four genes strongly associated with SB (FDR < 0.1) (Table 2). *SERPINF1* was the top gene linked to SB. Both the *nonsyn* (*p* = 1.3E-6) and the *exonreg* (*p* = 1.7E-6) variant groups of this gene remained genome wide significant after a multiple testing correction. Four out of the nine *nonsyn* rare variants in *SERPINF1* had C-scores between 23 and 29, representing between 0.1–0.5% most deleterious mutations. One of the four probably damaging variants, rs1804145, was also suggestively associated with SB-AUD in the bivariate analysis (*p* = 1.1E-7, see Table 1). Additional genes significantly associated with SB included: *ZNF30, CD34*, *SLC5A9, OPRD1*, and *HSD17B3*. A lincRNA *AC002511*.*3* is also associated with SB. Of the 15 *nonsyn* variants on *ZNF30*, one has a C-score of 33 (~ 0.05% most deleterious), and six have C-scores between 22 and 24 (~ 0.5% most deleterious). One of the nine *nonsyn* variants on *CD34* has a C-score of 32. Seven out of the 12 *nonsyn* variants on *SCL5A9* have C-scores ranging from 24 to 30. *OPRD1* has three rare exon variants, one of which has a C-score of 18 (1.6% most deleterious), and one of the 10 rare variants on *HSD17B3* has a C-score of 22.8 (~ 0.5% most deleterious).

**Figure 3:**
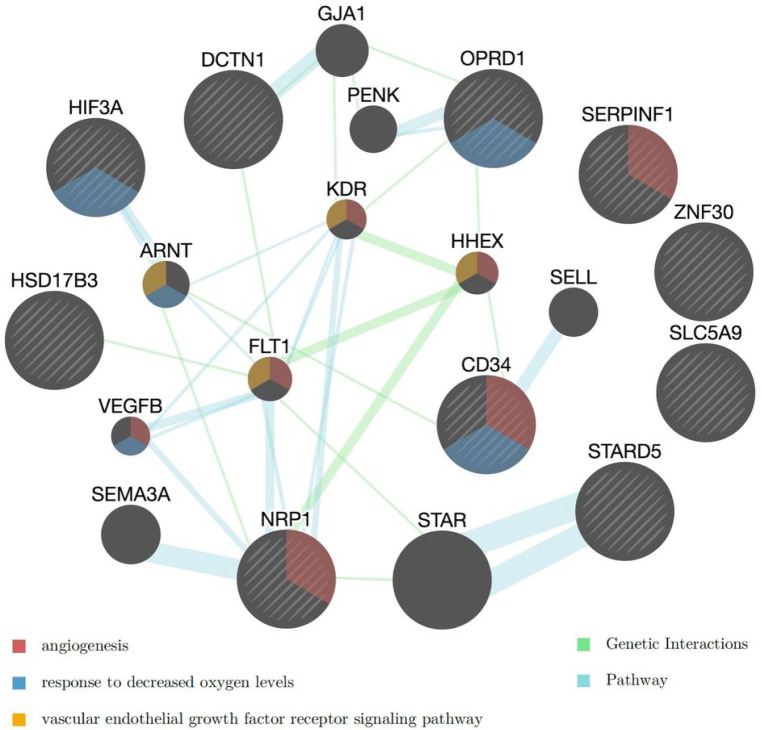
Networks of the rare-variant genes associated with suicidal behaviors in AI. Three of the enriched functional networks are highlighted in the gene nodes (see Table S4 for the complete list). Yellow: vascular endothelial growth factor (VEGF) receptor signaling pathway (FDR=2.1E-04); Red: angiogenesis (FDR=2.1E-04); Blue: response to decreased oxygen levels (FDR=4.2E-03). The color of the edges linking the genes indicate the type of database used in the GeneMania analysis. Blue: Pathways; Green: Genetic interactions.

A gene set enrichment analysis for the 11 genes whose rare and low frequency variants were associated with SB at FDR < 0.1 (Table 2) identified axon to be the most significantly enriched gene ontology term (nominal *p* = 1.63E-5, adjusted *p* = 0.016). Four of the 11 genes belong to the axon gene set: *OPRD1*, *NRP1*, *SERPINF1*, and *DCTN1*. Network analysis conducted using GeneMania for the 11 genes in Table 2 recognized 10 genes and automatically selected 10 additional related genes (Fig. 3). The analysis considered pathways and genetic interactions and identified a number of significantly associated functional networks (Table S4). Figure 3 illustrates the top enriched distinct functions, including VEGF signaling (FDR = 2.1E-04), angiogenesis (FDR = 2.1E-04), and response to decreased oxygen levels (FDR = 4.2E-03). VEGF, as an agngiogenic cytokine, and hypoxia have been associated with depression and suicide (64, 65).

### 3.3. Pathways with rare and low-frequency variants associated with suicidal behaviors in AI

Regulation of gene expression by hypoxia inducible factor (HIF) is the top pathway (*p* = 1.4E-5) whose rare and low-frequency variants were associated with SB (Table 3). The pathway is comprised of 10 genes with total of 91 nonsynonymous or splicing site variants (*nonysn*), of which 83 have MAF < 5% and were included in the association test. Hypoxia could decrease serotonin synthesis, which has been linked to suicide (66). The three additional pathways that were associated with SB at FDR < 0.1 are essentially two distinct pathways: vasopressin regulated water reabsorption with 164 *nonsyn* rare variants on 44 genes, and cellular hexose transport with 126 *nonsyn* rare variants on 21 genes. Vasopressin plays a critical role in regulating renal water reabsorption and cardiovascular homeostasis. As an integral part of the hypothalamic-pituitary-adrenal (HPA) axis, it is also an important player in response to stress and contributes to stress-related disorders such as anxiety and depression (67). The cellular hexose transport pathway includes gene families responsible for glucose transport in humans. Sodium independent glucose transporters (GLUTs) are encoded by the SLC2 family, while sodium dependent glucose transporters (SGLTs) are encoded by the SLC5 family.

## 4. DISCUSSION

Despite unprecedented strategies to support its prevention as a public health imperative, suicide has, alarmingly, increased in recent years—now representing the second leading cause of death in teens and young adults in the U.S. as well as worldwide (68, 69). American Indians and Alaska Natives (AI/AN) demonstrate the highest burden of suicide compared to all other ethnic or racial groups, highlighting urgency to critically advance research and prevention (3–6). However, rates of suicide vary substantially between tribal groups and by distinct geographic region (2), underscoring the need to delineate risk and resilience factors in local communities to develop community-specific prevention and intervention efforts (10–12, 70).

The present study focused on an American Indian population living on eight contiguous reservations. This population demonstrates high rates of suicide as well as AUD (19, 43). In this study, we investigated potential genetic risk factors for suicidal behaviors by studying: (1) the possible genetic overlap of SB with AUD, and (2) the impacts of rare- and low-frequency genomic variants on SB. We identified five variants significantly associated with SB-AUD, two of which are intergenic and three are intronic. Two of the five variants, although not significantly associated with either SB or AUD severity on their own in this AI cohort, were significantly associated with the dual phenotype SB-AUD—suggesting the genetic correlations were induced by residual shared factors. Six genes, one lincRNA, and one canonical pathway, were found to be significantly associated with SB through rare and low-frequency variant analysis. Four additional genes, and two pathways, also were strongly associated with SB.

### **F-box gene** FBXO11 **significantly associated with SB-AUD in this AI population**

The intronic variants significantly associated with SB-AUD are on genes *AACSP1*, *ANK1*, and *FBXO11*. *AACSP1* is a pseudogene whose function is unclear, although it has been associated with an Alzheimer’s marker in an interaction analysis (71). *ANK1* encodes ankyrin-1 protein active in red blood cells as well as brain and muscle. The gene has been linked to Alzheimer’s disease through epigenetic deregulation (72, 73) and appears to play a role in immunomodulation (74).

Rs184204326 on gene *FBXO11* was the only common variant significantly associated with SB-AUD. *FBXO11* encodes a member of the F-box protein family and is highly conserved in evolution. It is part of the SCF (SKP1-cullin-F-box) complex, which is responsible for ubiquitination and degradation of SCF substrates and plays an important role in the maintenance of genome stability (75). *FBXO11* is also involved in regulating alternative splicing (76). While the gene is expressed in various tissues, it is particularly abundant in the brain. *FBXO11* has not been previously associated with SB. However, variants on or near *FBXO11* have been associated with a number or correlated behavior traits and disorders such as alcohol consumption (77), smoking initiation (77, 78), risk taking behaviors (79, 80), externalizing behaviors (81), educational attainment (82), insomnia (83), schizophrenia and depression (84, 85). *De novo* mutations on *FBXO11* have also been found to cause intellectual disability with behavior problems as well as facial dysmorphisms (86) and other neurodevelopmental disorders (87). Variant rs77969729 on *FBXO11* has been linked to Alzheimer’s disease in the UKBiobank (88). This variant is in LD with rs184204326 on *FBXO11* in the AI cohort and associated with SB-AUD at *p* = 3.4E-7.

### Rare mutations in PEDF are likely a risk factor for suicidal behaviors

Six genes and one lincRNA were found to be significantly associated with SB through rare variant analysis. The top gene *SERPINF1* encodes serpin F1, also known as pigment epithelium-derived factor (PEDF). This gene has nine rare nonsynonymous or splicing site mutations, of which four are among the 0.1–0.5% most deleterious. Nearly 6% of individuals in the AI cohort carry some of these mutations. PEDF is a secreted glycoprotein and a potent inhibitor of angiogenesis (89). It also has neuroprotective effects and been implicated in depression (90, 91). Reduction in PEDF levels have been found in the plasmas of patients with major depressive disorder (MDD), as well as in the prefrontal cortex (PFC) of animal models exhibiting depressive-like behaviors (91). Conversely, overexpression of PEDF in the PFC has been shown to induce antidepressant “like” behaviors, by exerting effects on the tryptophan and glutamate in the PFC, with tryptophan being an essential amino acid precursor of serotonin, which is known to be associated with both depression and SB (92). Another study has shown that PEDF in the hippocampus has a similar effect on depressive phenotypes in animal models by contributing to the synaptic formation and Wnt signaling activation in that region (93). PEDF has thus been suggested as a biomarker and a novel therapeutic target for depression (90).

Additional genes significantly associated with SB in the AI cohort included *ZNF30*, *CD34*, *SLC5A9*, *OPRD1*, and *HSD17B3*. *ZNF30* encodes a zinc finger protein. A microdeletion of five genes including ZNF30 results in chromosome 19q13.11 deletion syndrome that includes features such as: developmental delay, microcephaly and intellectual disabilities (94). The GWAS meta-analysis by the international suicide genetics consortium (ISGC) reported another zinc finger family gene (*ZNF28*) associated with SB and suicide death (31). *CD34* is involved in the innate immune system. Its variants have been associated with Alzheimer’s disease (88) and risk-taking behavior (80). *SCL5A9* is involved in sodium ion transport and is also known to be a sodium-dependent glucose transporter (95). This gene has been associated with amygdala volume (96) and metabolic measurements (97). Variants on or near *HSD17B3* have been linked to smoking behavior (98), brain morphology measurements (99), memory performance (100) and Alzheimer’s markers (71, 101).

Opioid receptor delta 1 (*OPRD1*) gene encodes a member of the opioid family of the G-protein coupled receptors (GPCR). Delta opioid receptors are involved in reward mediation and neuroprotection. *OPRD1* is specifically involved in the opioid receptor signaling pathway and cellular response to hypoxia. Numerous candidate gene studies have implicated *OPRD1* in addiction, including opioid, cocaine, and alcohol dependence (102, 103). Variants on *OPRD1* have also been associated with schizophrenia (104) and educational attainment (82).

### Hypoxia regulation is significantly associated with suicidal behaviors

*Nonsyn* rare variants in a pathway related to hypoxia inducible factor (HIF) regulation were significantly associated with SB (see Table 3). Nearly half of the individuals in the AI cohort have some of these rare mutations on their genes in this pathway. The network analysis of the top rare-variant genes associated with SB also found that those genes were enriched for response to decreased oxygen levels (see Fig. 3).

Chronic hypoxia is suggested to be a risk factor for suicide (64), and metabolic stress associated with hypoxia is a possible mechanism (105). Hypoxia is also hypothesized to increase the risk of suicide by reducing the synthesis of brain serotonin (66) or downregulating PEDF (106), which has a protective role in depression and is associated with SB in this AI cohort. HIFs are transcriptional factors that respond to reduced oxygen levels in cell and tissue. HIF-1 protects against hypoxia and reduces oxidative stress (107), while HIF-2α plays an important in the modulation of inflammatory responses (108). Recent studies have indicated that oxidative stress and abnormal energy metabolism in the brain play significant roles in the development of depression. Therefore, increasing HIF-1 activity has been suggested as a potential new therapeutic target for depression and suicide ideation (107). Gene expression analyses have demonstrated that patients with MDD exhibit increased expression of HIF-1 and its target genes, including *VEGF* and *GLUT1* (*SLC2A1*) (65). Our network analysis of the top rare-variant genes associated with SB in the AI have also found enrichments in VEGF receptors signaling and angiogenesis.

Alcohol exposure also can alter expression of HIF. For instance, alcohol exposure can induce HIF-1α activation, however the dose and timing of alcohol exposure results in differential expression of HIF-1α in the brain and other organs (109). Both acute and chronic alcohol exposure have been found to increase HIF-1α expression in the brain cortex, whereas chronic binge alcohol exposure decreased HIF-1α expression (109). Another study found HIF3A can be epigenetically induced in the amygdala in animal models by acute alcohol exposure, and its epigenetic reprogramming was associated with the anti-anxiety effect of acute alcohol exposure (110). *Nonsyn* rare variants in *HIF3A* were strongly associated with SB (nominal *p* = 2.9E-05, FDR = 0.09) in the present study.

### Vasopressin regulated water metabolism and hexose transport strongly associated with suicidal behaviors

*Nonsyn* rare variants in two additional pathways, vasopressin-regulated water reabsorption and cellular hexose transport, were found to be strongly associated with SB in the AI (FDR < 0.1). Vasopressin is an evolutionary ancient neuropeptide that is involved in regulating physiological processes such as renal water reabsorption and cardiovascular homeostasis. It also plays an important role in the modulation of emotional and social behaviors in the brain (111, 112), with vasopressin containing neurons most abundantly found in the hypothalamus. The vasopressin system is known to interact with the HPA axis (67). Indeed, cortisol response and stress reactivity within the HPA axis is well-established as an endophenotype for depression and SB, as increased cortisol level is associated with death by suicide (113, 114). HPA axis dysfunction has also been observed in those with a history of suicide attempts (114). In addition, changes in water and electrolyte metabolism have been reported in clinical studies of depressed patients (115, 116). Alcohol also uniquely interacts with the vasopressin system. Alcohol is a diuretic that promotes water loss by inhibiting the production of vasopressin. The HPA axis response to alcohol can be altered by manipulating the vasopressin system (117). The vasopressin system has thus become an emerging therapeutic target for stress and depression, as well as alcohol-related behaviors (67, 117). Gene families in the cellular hexose transport pathway mediate glucose absorption in the small intestine, glucose reabsorption in the kidney, glucose uptake by the brain across the blood-brain barrier, and glucose release by all cells in the body (118). Evidence suggests that disturbances in glucose metabolism may be associated with suicidal ideation and attempts (119). A recent study has found a significant association between blood glucose and suicide attempts in male patients with MDD (120). Taken together, our findings suggest that pathways underlying vasopressin-regulated water reabsorption and the cellular hexose transport system warrant additional investigation in association with SB.

### Strengths and Limitations

The results of this study should be interpreted in light of several limitations. The analyses were not meant to generate a comprehensive model of suicide risk and AUD in this community group, but rather to determine whether specific genetic associations could be identified for suicidal thoughts and acts, and between SB and AUD phenotypes using genetic analyses. A larger sample, powered to access and assess additional variables associated with suicide risk, particularly according to the above systems, is recommended. Next, our findings may not generalize to other American Indians in the population from which the sample was drawn or be representative of all American Indians, as rates of AUD and suicide vary among tribes (17, 19, 121). In addition, we used retrospective data for lifetime measures of suicide risk, which are subject to recall bias and may include reporting bias for psychiatric symptoms. Lifetime suicidal ideation and behaviors were assessed using a subscale of the SSAGA as well as verified death records. Prospective investigations, using validated measures of suicidal symptom severity and intensity of suicidal ideation, and indexing the severity and lethality of SB, are recommended to further elucidate specific symptom relationships. Despite these limitations and in consideration of the current sample size, we were able to identify several genes and pathways implicated in SB and AUD severity. Our findings suggest that rare variant analysis in a high-risk population, enabled by whole-genome sequencing, has the potential to identify novel genetic factors influencing suicidal behaviors. Bivariate association analysis between comorbid disorders may further increase statistical power when the same loci potentially contribute to both disorders.

In conclusion, we conducted the first genome-wide bivariate association analysis for SB and AUD, and identified five genome-wide significant loci. We also conducted the first large-scale rare variant analysis and identified 11 novel genes and three pathways for SB. Of particular importance, this study represents the first investigation of genetic factors for SB in an American Indian population that has high risk for suicide. Although our findings may be population specific, the rare functional mutations relating to PEDF and HIF regulation may suggest an important mechanism underlying suicidal behaviors and a potential therapeutic target for treatment and intervention in the prevention of suicide.

## Figures and Tables

**Table 1:** Top variants identified in bivariate genome-wide associations for suicidal behaviors and AUD severity in the American Indians.

Chr:pos	dbSNP ID	Geno^1^	Gene	Location	MAF	C-score^2^	β_AUD_^3^	β_SB_	*P*_AUD_^4^	*P*_SB_	*P*_SB–AUD_^5^
13:100709891	-	C/T	*ZIC2-PCCA*	Intergenic	0.024	1.19	17.2	0.8	2.48E-08	3.63E-05	**2.87E-09**
4:163743605	rs200577368	G/T	*NAF1-FSTL5*	Intergenic	0.026	0.02	16.9	0.6	2.52E-09	1.33E-03	**3.17E-09**
5:178238561	rs76300969	G/A	*AACSL/AACSP1*	Intron	0.010	10.03	−5.7	1.6	2.37E-01	2.08E-07	**4.16E-09**
8:41731811	rs530542541	A/C	*ANK1*	Intron	0.044	0.76	12.7	0.5	4.91E-08	4.61E-04	**2.22E-08**
2:48105656	rs184204326	G/C	*FBXO11*	Intron	0.066	0.28	0.5	0.7	7.77E-01	1.48E-07	**3.63E-08**
9:22528934	rs79833306	C/T	*DMRTA1*	Downstream	0.031	13.32	15.2	0.4	1.95E-08	1.14E-02	6.75E-08
1:169344460	-	C/T	*BLZF1*	Intron	0.035	5.71	13.5	0.5	9.46E-08	2.45E-03	1.06E-07
17:1674434	rs1804145	C/G	*SERPINF1*	Exon	0.011	22.80	−3.3	1.4	4.56E-01	3.05E-07	1.12E-07
17:1683043	rs12951668	C/T	*SMYD4*	3’UTR	0.011	2.18	−3.3	1.4	4.56E-01	3.05E-07	1.12E-07
14:84263845	rs142827459	C/T	*BX248253*	Downstream	0.028	0.44	8.9	1.0	2.52E-03	8.01E-08	1.58E-07

1 Genotype: reference/alternative.

2 C-score: CADD score indicating how likely a variant to be deleterious. The higher the score the more likely.

3 β_AUD_: beta for AUD severity; β_SB_: beta for suicidal behaviors (SB).

4 *P*_AUD_: *p*-value for AUD severity univariate association; *P*_SB_: *p*-value for SB univariate association.

5 *P*_SB–AUD_: *p*-value for SB and AUD severity bivariate association. Bold font: genome-wide significant.

**Table 2:** Top genes with rare and low-frequency variants associated with suicidal behaviors in the American Indians.

Chr	Positions	Genes^1^	Variants^2^	SNPs^3^	C-scores^4^	%Rare^5^	*p*-value^6^	FDR^7^
17	1673263–1680721	SERPINF1	Nonsyn	9 (10)	0-4-0	5.5	1.32E-06	**0.024**
17	1665330–1680847	SERPINF1	ExonReg	12 (17)	1-4-0	5.9	1.65E-06	**0.024**
19	35418193–35435632	ZNF30	Nonsyn	15 (21)	3-6-1	8.5	1.76E-06	**0.024**
1	208062056–208073330	CD34	Nonsyn	9 (12)	2-0-1	2.7	3.55E-06	**0.032**
19	35923858–35924757	AC002511.3	ExonReg	3 (5)	0-0-0	2.6	3.89E-06	**0.032**
1	48694570–48713100	SLC5A9	Nonsyn	12 (16)	1-7-0	8.7	6.44E-06	**0.044**
1	29138936–29190138	OPRD1	ExonReg	3 (7)	1-0-0	3.1	7.44E-06	**0.044**
9	98997810–99064425	HSD17B3	ExonReg	10 (12)	0-1-0	10.4	9.20E-06	**0.047**
1	48688376–48714188	SLC5A9	ExonReg	27 (35)	3-7-0	20.2	1.03E-05	**0.047**
2	74588717–74598791	*DCTN1*	Nonsyn	11 (11)	2-7-1	9.6	1.73E-05	0.072
15	81605656–81614811	*STARD5*	Nonsyn	3 (3)	2-1-0	5.7	2.39E-05	0.087
10	33475282–33552763	*NRP1*	Nonsyn	9 (12)	2-4-0	8.6	2.53E-05	0.087
19	46807185–46832554	*HIF3A*	Nonsyn	11 (14)	1-5-0	14.1	2.89E-05	0.092

1 Bold font: gene is significantly associated with suicidal behaviors at the genome level after multiple comparison correction.

2 Variant groups: ExonReg includes variants on exon and upstream/downstream of a gene; Nonsyn includes nonsynonymous and splicing variants of a gene.

3 Number of rare/low-frequency markers included in the test for each gene. The number in the parenthesis is the total number of SNPs of the same category on the gene.

4 C-scores counts: three numbers c1-c2-c3 represent the number of rare variants that have CADD scores (C-scores) in the range of 15–20 (1–3% most deleterious mutations), 20–30 (0.1–1% most deleterious), and > 30 (< 0.1% most deleterious). For instance, the first row *SERPINF1* Nonsyn group has 9 variants. 0-4-0 indicates that 0 of the 9 SNPs has C-scores in the range of 15–20 or over 30, while 4 SNPs have C-scores between 20 and 30. The higher the C-score, the more likely a variant is deleterious.

5 The fraction of individuals that have at least one of the rare/low-frequency markers on the gene.

6 Nominal *p*-values.

7 FDR-adjusted *p*-values, combing the two gene variant groups (Number of genes tested in each group: ExonReg = 28718; Nonsyn = 12588). Bold font: genome-wide significance (FDR < 0.05).

**Table 3:** Top pathways or gene sets with rare and low-frequency nonsynonymous variants associated with suicidal behaviors (FDR < 0.1) in the American Indians.

Database	Pathway	SNPs^1^	%Rare^2^	*p*-value^3^	FDR^4^	Genes in pathway
Reactome	REGULATION OF GENE EXPRESSION BY HYPOXIA INDUCIBLE FACTOR	83 (91)	49.5	1.35E-05	0.040	*CREBBP,EP300,HIF1A,CA9,VEGFA,EPAS1(HIF2A),HIF3EPO,ARNT (HIF1B),CITED2,HIGD1A*
WikiPathways	VASOPRESSIN REGULATED WATER REABSORPTION	165 (190)	78.9	4.94E-05	0.055	*CREB5,DYNLL1,CREB1,DYNC1I2,DYNC1I1,ARHGDIA,DYNC1H1,AVP,ARHGDIB,DCTN4,NSF,ADCY6,ARHGDIG,AVPR2,DYNC1LI1,ADCY9,AQP2,DCTN6,DYNC2H1,VAMP2,DYNLL2,DCTN1,RAB5C,DYNC2L1,RAB11A,CREB3L1,CREB3L2,RAB11B,AQP3,AQP4,GNAS,DCTN5,DYNC1LI2,CREB3L3,CREB3L4,STX4,PRKACA,PRKACB,ADCY3,PRKACG,DCTN2,CREB3,RAB5A,RAB5B*
KEGG	VASOPRESSIN REGULATED WATER REABSORPTION	164 (189)	78.3	5.53E-05	0.055	*ADCY9,ADCY6,DYNLL2,DYNC1LI2,VAMP2,DCTN6,CREB3L4,AQP4,PRKX,RAB11A,DYNLL1,DCTN2,AQP3,CREB3L1,ADCY3,CREB3,RAB5B,AVPR2,RAB5A,CREB3L3,NSF,DCTN5,AVP,CREB3L2,RAB11B,STX4,PRKACA,PRKACB,PRKACG,CREB1,GNAS,DYNC2H1,DYNC1H1,DCTN4,DYNC1I2,DCTN1,DYNC1I1,CREB5,AQP2,DYNC2LI1,ARHGDIA,ARHGDIB,DYNC1LI1,RAB5C*
Reactome	CELLULAR HEXOSE TRANSPORT	126 (151)	58.0	1.17E-04	0.087	*SLC2A3,SLC5A1,SLC5A4,FGF21,SLC2A9,SLC2A1,SLC5A9,SLC2A11,SLC2A8,SLC5A2,SLC2A12,SLC5A10,SLC45A3,SLC2A6,SLC2A2,SLC50A1,MFSD4B,SLC2A14,SLC2A4,SLC2A7,SLC2A10*

1 Number of markers included in the test for each gene. The number in the parenthesis is the total number of nonsynonymous or splicing-site SNPs on the gene.

2 The fraction of individuals that have at least one of the rare/low-frequency markers on the gene.

3 Nominal *p*-values.

4 FDR-adjusted *p*-values. Total number of pathways tested is 2981.

## Data Availability

The data that support the finding of this study are available by contacting the last author. However, data availability is subject to approval of the specific AI tribes participating.

## References

[1] Curtin SC, Warner M, Hedegaard H. Increase in Suicide in the United States, 1999–2014. NCHS Data Brief. 2016(241):1–8. 27111185

[2] Department of Health and Human Services, Indian Health Service, Office of Public Health Support, Division of Program Statistics. Trends in Indian Health 2014. 2014:[Available from: https://www.ihs.gov/dps/publications/trends2014/.

[3] Karaye IM. Differential trends in US suicide rates, 1999–2020: Emerging racial and ethnic disparities. Preventive Medicine. 2022;159:107064. 35452714 10.1016/j.ypmed.2022.107064

[4] Leavitt R, Ertl A, Sheats K, Petrosky E, Ivey-Stephenson A, Fowler K. Suicides Among American Indian/Alaska Natives — National Violent Death Reporting System, 18 States, 2003–2014. MMWR Morb Mortal Wkly Rep. 2018;67:237–42. 29494572 10.15585/mmwr.mm6708a1 PMC5861703

[5] Wilson RF, Liu G, Lyons BH, Petrosky E, Harrison DD, Betz CJ, Surveillance for Violent Deaths — National Violent Death Reporting System, 42 States, the District of Columbia, and Puerto Rico, 2019. MMWR Surveillance Summaries. 2022;71(6):1–40. 10.15585/mmwr.ss7106a1 PMC9129903 35588398

[6] Bolton S-L, Elias B, Enns MW, Sareen J, Beals J, Novins DK. A comparison of the prevalence and risk factors of suicidal ideation and suicide attempts in two American Indian population samples and in a general population sample. Transcultural Psychiatry. 2013;51(1):3–22. 24065607 10.1177/1363461513502574 PMC4530965

[7] Chachamovich E, Kirmayer LJ, Haggarty JM, Cargo M, McCormick R, Turecki G. Suicide among Inuit: Results from a Large, Epidemiologically Representative Follow-Back Study in Nunavut. The Canadian Journal of Psychiatry. 2015;60(6):268–75. 26175324 10.1177/070674371506000605 PMC4501584

[8] Cwik M, Doty SB, Hinton A, Goklish N, Ivanich J, Hill K, Community Perspectives on Social Influences on Suicide Within a Native American Reservation. Qualitative Health Research. 2021;32(1):16–30. 34825619 10.1177/10497323211045646 PMC10040248

[9] Freedenthal S, Stiffman AR. Suicidal Behavior in Urban American Indian Adolescents: A Comparison with Reservation Youth in a Southwestern State. Suicide and Life-Threatening Behavior. 2004;34(2):160–71. 15191272 10.1521/suli.34.2.160.32789

[10] Livingston R, Daily RS, Guerrero APS, Walkup JT, Novins DK. No Indians to Spare: Depression and Suicide in Indigenous American Children and Youth. Child and Adolescent Psychiatric Clinics of North America. 2019;28(3):497–507. 31076123 10.1016/j.chc.2019.02.015

[11] Shaw JL, Beans JA, Comtois KA, Hiratsuka VY. Lived Experiences of Suicide Risk and Resilience among Alaska Native and American Indian People. International Journal of Environmental Research and Public Health [Internet]. 2019; 16(20). 10.3390/ijerph16203953 PMC6843805 31627325

[12] Trout L, Wexler L. Arctic Suicide, Social Medicine, and the Purview of Care in Global Mental Health. Health Hum Rights. 2020;22(1):77–89. 32669791 PMC7348442

[13] Indian Health Service. U S Indian Health Service, Division of Program Statistics: Trends in Indian Health. Rockville, MD: U S Department of Health and Human Services, Public Health Service, Indian Health Service; 1997.

[14] Spillane NS, Kirk-Provencher KT, Schick MR, Nalven T, Goldstein SC, Kahler CW. Identifying Competing Life Reinforcers for Substance Use in First Nation Adolescents. Substance Use & Misuse. 2020;55(6):886–95. 31965888 10.1080/10826084.2019.1710206 PMC7224337

[15] Compton WM, Thomas YF, Stinson FS, Grant BF. Prevalence, correlates, disability, and comorbidity of DSM-IV drug abuse and dependence in the United States: results from the national epidemiologic survey on alcohol and related conditions. Archives of General Psychiatry. 2007;64(5):566–76. 17485608 10.1001/archpsyc.64.5.566

[16] Brave Heart MYH, Lewis-Fernández R, Beals J, Hasin DS, Sugaya L, Wang S, Psychiatric disorders and mental health treatment in American Indians and Alaska Natives: results of the National Epidemiologic Survey on Alcohol and Related Conditions. Social Psychiatry and Psychiatric Epidemiology. 2016;51(7):1033–46. 27138948 10.1007/s00127-016-1225-4 PMC4947559

[17] Beals J, Novins DK, Whitesell NR, Spicer P, Mitchell CM, Manson SM. Prevalence of Mental Disorders and Utilization of Mental Health Services in Two American Indian Reservation Populations: Mental Health Disparities in a National Context. American Journal of Psychiatry. 2005;162(9):1723–32. 16135633 10.1176/appi.ajp.162.9.1723

[18] Spicer P, Beals J, Croy CD, Mitchell CM, Novins DK, Moore L, The prevalence of DSM-III-R alcohol dependence in two American Indian populations. Alcohol Clin Exp Res. 2003;27(11):1785–97. 14634495 10.1097/01.ALC.0000095864.45755.53

[19] Ehlers CL, Wall TL, Betancourt M, Gilder DA. The clinical course of alcoholism in 243 Mission Indians. American Journal of Psychiatry. 2004;161(7):1204–10. 15229052 10.1176/appi.ajp.161.7.1204

[20] Robin RW, Long JC, Rasmussen JK, Albaugh B, Goldman D. Relationship of Binge Drinking to Alcohol Dependence, Other Psychiatric Disorders, and Behavioral Problems in an American Indian Tribe. Alcohol: Clinical and Experimental Research. 1998;22(2):518–23. 9581662

[21] Grant BF, Goldstein RB, Saha TD, Chou SP, Jung J, Zhang H, Epidemiology of DSM-5 alcohol use disorder: Results from the national epidemiologic survey on alcohol and related conditions III. JAMA Psychiatry. 2015;72(8):757–66. 26039070 10.1001/jamapsychiatry.2015.0584 PMC5240584

[22] Kaplan MS, Huguet N, McFarland BH, Caetano R, Conner KR, Nolte KB, Heavy Alcohol Use Among Suicide Decedents: Differences in Risk Across Racial-Ethnic Groups. Psychiatric Services. 2016;67(3):258-. 26725300 10.1176/appi.ps.201500494

[23] Ehlers CL, Kim C, Gilder DA, Stouffer GM, Caetano R, Yehuda R. Lifetime history of traumatic events in a young adult Mexican American sample: Relation to substance dependence, affective disorder, acculturation stress, and PTSD. Journal of Psychiatric Research. 2016;83:79–85. 27569652 10.1016/j.jpsychires.2016.08.009 PMC5107155

[24] Cherpitel CJ, Borges GLG, Wilcox HC. Acute Alcohol Use and Suicidal Behavior: A Review of the Literature. Alcohol: Clinical and Experimental Research. 2004;28(s1):18S–28S. 10.1097/01.alc.0000127411.61634.14 15166633

[25] Kaplan MS, Giesbrecht N, Caetano R, Conner KR, Huguet N, McFarland BH, Acute Alcohol Consumption as a Contributing Factor to Suicidal Behavior. American Journal of Public Health. 2013;103(9):e2–e3. 10.2105/AJPH.2013.301422 PMC3780687 23865652

[26] Caetano R, Kaplan MS, Kerr W, McFarland BH, Giesbrecht N, Kaplan Z. Suicide, Alcohol Intoxication, and Age Among Whites and American Indians/Alaskan Natives. Alcohol: Clinical and Experimental Research. 2020;44(2):492–500. 10.1111/acer.14251 PMC7018549 31782530

[27] Edenberg HJ, Gelernter J, Agrawal A. Genetics of Alcoholism. Current Psychiatry Reports. 2019;21(4):26. 30852706 10.1007/s11920-019-1008-1

[28] Erlangsen A, Appadurai V, Wang Y, Turecki G, Mors O, Werge T, Genetics of suicide attempts in individuals with and without mental disorders: a population-based genome-wide association study. Molecular Psychiatry. 2020;25(10):2410–21. 30116032 10.1038/s41380-018-0218-y PMC7515833

[29] Stephenson M, Lannoy S, Edwards AC. Shared genetic liability for alcohol consumption, alcohol problems, and suicide attempt: Evaluating the role of impulsivity. Translational Psychiatry. 2023;13(1):87. 36899000 10.1038/s41398-023-02389-3 PMC10006209

[30] Docherty AR, Mullins N, Ashley-Koch AE, Qin XJ, Coleman J, Shabalin AA, Genome-wide association study meta-analysis of suicide attempt in 43,871 cases identifies twelve genome-wide significant loci. medRxiv. 2022:2022.07.03.22277199.

[31] Li QS, Shabalin AA, DiBlasi E, Gopal S, Canuso CM, Palotie A, Genome-wide association study meta-analysis of suicide death and suicidal behavior. Molecular Psychiatry. 2023;28(2):891–900. 36253440 10.1038/s41380-022-01828-9 PMC9908547

[32] Mullins N, Kang J, Campos AI, Coleman JRI, Edwards AC, Galfalvy H, Dissecting the Shared Genetic Architecture of Suicide Attempt, Psychiatric Disorders, and Known Risk Factors. Biological Psychiatry. 2022;91(3):313–27. 34861974 10.1016/j.biopsych.2021.05.029 PMC8851871

[33] Kimbrel NA, Ashley-Koch AE, Qin XJ, Lindquist JH, Garrett ME, Dennis MF, A genome-wide association study of suicide attempts in the million veterans program identifies evidence of pan-ancestry and ancestry-specific risk loci. Molecular Psychiatry. 2022;27(4):2264–72. 35347246 10.1038/s41380-022-01472-3 PMC9910180

[34] Strawbridge RJ, Ward J, Ferguson A, Graham N, Shaw RJ, Cullen B, Identification of novel genome-wide associations for suicidality in UK Biobank, genetic correlation with psychiatric disorders and polygenic association with completed suicide. eBioMedicine. 2019;41:517–25. 30745170 10.1016/j.ebiom.2019.02.005 PMC6442001

[35] Fu Q, Heath AC, Bucholz KK, Nelson EC, Glowinski AL, Goldberg J, A twin study of genetic and environmental influences on suicidality in men. Psychological Medicine. 2002;32(1):11–24. 11883722 10.1017/s0033291701004846

[36] Voracek M, Loibl LM. Genetics of suicide: a systematic review of twin studies. Wiener klinische Wochenschrift. 2007;119(15):463–75. 17721766 10.1007/s00508-007-0823-2

[37] Ruderfer DM, Walsh CG, Aguirre MW, Tanigawa Y, Ribeiro JD, Franklin JC, Significant shared heritability underlies suicide attempt and clinically predicted probability of attempting suicide. Molecular Psychiatry. 2020;25(10):2422–30. 30610202 10.1038/s41380-018-0326-8 PMC6609505

[38] Peng Q, Ehlers CL. Long tracks of homozygosity predict the severity of alcohol use disorders in an American Indian population. Molecular Psychiatry. 2021. 10.1038/s41380-020-00989-9 PMC8254832 33398086

[39] Kryukov GV, Pennacchio LA, Sunyaev SR. Most Rare Missense Alleles Are Deleterious in Humans: Implications for Complex Disease and Association Studies. The American Journal of Human Genetics. 2007;80(4):727–39. 17357078 10.1086/513473 PMC1852724

[40] DiBlasi E, Shabalin AA, Monson ET, Keeshin BR, Bakian AV, Kirby AV, Rare protein-coding variants implicate genes involved in risk of suicide death. American Journal of Medical Genetics Part B: Neuropsychiatric Genetics. 2021;186(8):508–20. 10.1002/ajmg.b.32861 PMC9292859 34042246

[41] Ehlers CL, Gilder DA, Gizer IR, Wilhelmsen KC. Indexing the ‘dark side of addiction’: substance-induced affective symptoms and alcohol use disorders. Addiction. 2019;114(1):139–49. 30153346 10.1111/add.14431 PMC6320236

[42] Ehlers CL, Gizer IR. Evidence for a genetic component for substance dependence in Native Americans. American Journal of Psychiatry. 2013;170(2):154–64. 23377636 10.1176/appi.ajp.2012.12010113 PMC3603686

[43] Ehlers CL, Yehuda R, Gilder DA, Bernert R, Karriker-Jaffe KJ. Trauma, historical trauma, PTSD and suicide in an American Indian community sample. Journal of Psychiatric Research. 2022;156:214–20. 36265258 10.1016/j.jpsychires.2022.10.012 PMC9842016

[44] Peng Q, Gizer IR, Libiger O, Bizon C, Wilhelmsen KC, Schork NJ, Association and ancestry analysis of sequence variants in ADH and ALDH using alcohol-related phenotypes in a Native American community sample. American Journal of Medical Genetics Part B: Neuropsychiatric Genetics. 2014;165(8):673–83. 10.1002/ajmg.b.32272 PMC4364382 25270064

[45] Peng Q, Gizer IR, Wilhelmsen KC, Ehlers CL. Associations Between Genomic Variants in Alcohol Dehydrogenase Genes and Alcohol Symptomatology in American Indians and European Americans: Distinctions and Convergence. Alcoholism: Clinical and Experimental Research. 2017;41(10):1695–704. 28815635 10.1111/acer.13480 PMC5626638

[46] Peng Q, Bizon C, Gizer IR, Wilhelmsen KC, Ehlers CL. Genetic loci for alcohol-related life events and substance-induced affective symptoms: indexing the “dark side” of addiction. Translational Psychiatry. 2019;9(1):71. 30718457 10.1038/s41398-019-0397-6 PMC6362044

[47] Peng Q, Wilhelmsen KC, Ehlers CL. Common genetic substrates of alcohol and substance use disorder severity revealed by pleiotropy detection against GWAS catalog in two populations. Addiction Biology. 2020:e12877. 32027075 10.1111/adb.12877 PMC7415504

[48] Schuckit MA. The Clinical Implications of Primary Diagnostic Groups Among Alcoholics. Archives of General Psychiatry. 1985;42(11):1043–9. 4051681 10.1001/archpsyc.1985.01790340021003

[49] Bucholz KK, Cadoret R, Cloninger CR, Dinwiddie SH, Hesselbrock VM, Nurnberger JI Jr., A new, semi-structured psychiatric interview for use in genetic linkage studies: a report on the reliability of the SSAGA. Journal of Studies on Alcohol. 1994;55(2):149–58. 8189735 10.15288/jsa.1994.55.149

[50] Schuckit MA, Smith TL, Anthenelli R, Irwin M. Clinical course of alcoholism in 636 male inpatients. American Journal of Psychiatry. 1993;150(5):786–92. 8480826 10.1176/ajp.150.5.786

[51] Bizon C, Spiegel M, Chasse SA, Gizer IR, Li Y, Malc EP, Variant calling in low-coverage whole genome sequencing of a Native American population sample. BMC Genomics. 2014;15(1):85. 24479562 10.1186/1471-2164-15-85 PMC3914019

[52] Allison DB, Thiel B, St. Jean P, Elston RC, Infante MC, Schork NJ. Multiple Phenotype Modeling in Gene-Mapping Studies of Quantitative Traits: Power Advantages. The American Journal of Human Genetics. 1998;63(4):1190–201. 9758596 10.1086/302038 PMC1377471

[53] Zhou X, Stephens M. Genome-wide efficient mixed-model analysis for association studies. Nat Genet. 2012;44(7):821–4. 22706312 10.1038/ng.2310 PMC3386377

[54] Lee S, Emond MJ, Bamshad MJ, Barnes KC, Rieder MJ, Nickerson DA, Optimal unified approach for rare-variant association testing with application to small-sample case-control whole-exome sequencing studies. American journal of human genetics. 2012;91(2):224–37. 22863193 10.1016/j.ajhg.2012.06.007 PMC3415556

[55] Kang HM, Sul JH, Service SK, Zaitlen NA, Kong S-Y, Freimer NB, Variance component model to account for sample structure in genome-wide association studies. Nature Genetics. 2010;42:348–54. 20208533 10.1038/ng.548 PMC3092069

[56] Subramanian A, Tamayo P, Mootha VK, Mukherjee S, Ebert BL, Gillette MA, Gene set enrichment analysis: A knowledge-based approach for interpreting genome-wide expression profiles. Proceedings of the National Academy of Sciences. 2005;102(43):15545. 10.1073/pnas.0506580102 PMC1239896 16199517

[57] Liberzon A, Birger C, Thorvaldsdóttir H, Ghandi M, Mesirov Jill P, Tamayo P. The Molecular Signatures Database Hallmark Gene Set Collection. Cell Systems. 2015;1(6):417–25. 26771021 10.1016/j.cels.2015.12.004 PMC4707969

[58] Rentzsch P, Schubach M, Shendure J, Kircher M. CADD-Splice—improving genome-wide variant effect prediction using deep learning-derived splice scores. Genome Medicine. 2021;13(1):31. 33618777 10.1186/s13073-021-00835-9 PMC7901104

[59] Kircher M, Witten DM, Jain P, O’Roak BJ, Cooper GM, Shendure J. A general framework for estimating the relative pathogenicity of human genetic variants. Nature Genetics. 2014;46(3):310–5. 24487276 10.1038/ng.2892 PMC3992975

[60] Watanabe K, Taskesen E, van Bochoven A, Posthuma D. Functional mapping and annotation of genetic associations with FUMA. Nature Communications. 2017;8(1):1826. 10.1038/s41467-017-01261-5 PMC5705698 29184056

[61] Warde-Farley D, Donaldson SL, Comes O, Zuberi K, Badrawi R, Chao P, The GeneMANIA prediction server: biological network integration for gene prioritization and predicting gene function. Nucleic Acids Research. 2010;38(suppl 2):W214–W20. 20576703 10.1093/nar/gkq537 PMC2896186

[62] Ramasamy A, Trabzuni D, Guelfi S, Varghese V, Smith C, Walker R, Genetic variability in the regulation of gene expression in ten regions of the human brain. Nat Neurosci. 2014;17(10):1418–28. 25174004 10.1038/nn.3801 PMC4208299

[63] Macpherson T, Hikida T. Role of basal ganglia neurocircuitry in the pathology of psychiatric disorders. Psychiatry and Clinical Neurosciences. 2019;73(6):289–301. 30734985 10.1111/pcn.12830

[64] Riblet NB, Gottlieb DJ, Watts BV, Cornelius SL, Fan VS, Shi X, Hypoxia-related risk factors for death by suicide in a national clinical sample. Psychiatry Research. 2019;273:247–51. 30658209 10.1016/j.psychres.2019.01.040 PMC8801295

[65] Mariani N, Cattane N, Pariante C, Cattaneo A. Gene expression studies in Depression development and treatment: an overview of the underlying molecular mechanisms and biological processes to identify biomarkers. Translational Psychiatry. 2021;11(1):354. 34103475 10.1038/s41398-021-01469-6 PMC8187383

[66] Young SN. Elevated incidence of suicide in people living at altitude, smokers and patients with chronic obstructive pulmonary disease and asthma: possible role of hypoxia causing decreased serotonin synthesis. Journal of Psychiatry and Neuroscience. 2013;38(6):423. 24148847 10.1503/jpn.130002 PMC3819157

[67] Morales-Medina JC, Witchey SK, Caldwell HK. The Role of Vasopressin in Anxiety and Depression. In: López-Muñoz F, Srinivasan V, de Berardis D, Álamo C, Kato TA, editors. Melatonin, Neuroprotective Agents and Antidepressant Therapy. New Delhi: Springer India; 2016. p. 667–85.

[68] Hedegaard H, Curtin SC, Warner M. Suicide Mortality in the United States, 1999–2019. NCHS Data Brief. 2021(398):1–8. 33663651

[69] The National Institute of Mental Health. Strategic Plan for Research, 20-MH-8096 2020. Available from: https://www.nimh.nih.gov/sites/default/files/documents/about/strategic-planning-reports/2020_nimh_strategic_plan_508.pdf.

[70] Cwik MF, O’Keefe VM, Haroz EE. Suicide in the pediatric population: screening, risk assessment and treatment. International Review of Psychiatry. 2020;32(3):254–64. 31922455 10.1080/09540261.2019.1693351 PMC7190447

[71] Wang H, Yang J, Schneider JA, De Jager PL, Bennett DA, Zhang H-Y. Genome-wide interaction analysis of pathological hallmarks in Alzheimer’s disease. Neurobiology of Aging. 2020;93:61–8. 32450446 10.1016/j.neurobiolaging.2020.04.025 PMC9795865

[72] Lunnon K, Smith R, Hannon E, De Jager PL, Srivastava G, Volta M, Methylomic profiling implicates cortical deregulation of ANK1 in Alzheimer’s disease. Nature Neuroscience. 2014;17(9):1164–70. 25129077 10.1038/nn.3782 PMC4410018

[73] De Jager PL, Srivastava G, Lunnon K, Burgess J, Schalkwyk LC, Yu L, Alzheimer’s disease: early alterations in brain DNA methylation at ANK1, BIN1, RHBDF2 and other loci. Nature Neuroscience. 2014;17(9):1156–63. 25129075 10.1038/nn.3786 PMC4292795

[74] Liscovitch N, French L. Differential Co-Expression between α-Synuclein and IFN-γ Signaling Genes across Development and in Parkinson’s Disease. PLOS ONE. 2014;9(12):e115029. 25493648 10.1371/journal.pone.0115029 PMC4262449

[75] Silverman JS, Skaar JR, Pagano M. SCF ubiquitin ligases in the maintenance of genome stability. Trends in Biochemical Sciences. 2012;37(2):66–73. 22099186 10.1016/j.tibs.2011.10.004 PMC3278546

[76] Yang Y, Hadjikyriacou A, Xia Z, Gayatri S, Kim D, Zurita-Lopez C, PRMT9 is a Type II methyltransferase that methylates the splicing factor SAP145. Nature Communications. 2015;6(1):6428. 10.1038/ncomms7428 PMC4351962 25737013

[77] Saunders GRB, Wang X, Chen F, Jang S-K, Liu M, Wang C, Genetic diversity fuels gene discovery for tobacco and alcohol use. Nature. 2022;612(7941):720–4. 36477530 10.1038/s41586-022-05477-4 PMC9771818

[78] Liu M, Jiang Y, Wedow R, Li Y, Brazel DM, Chen F, Association studies of up to 1.2 million individuals yield new insights into the genetic etiology of tobacco and alcohol use. Nature Genetics. 2019;51(2):237–44. 30643251 10.1038/s41588-018-0307-5 PMC6358542

[79] Baselmans B, Hammerschlag AR, Noordijk S, Ip H, van der Zee M, de Geus E, The Genetic and Neural Substrates of Externalizing Behavior. Biological Psychiatry Global Open Science. 2022;2(4):389–99. 36324656 10.1016/j.bpsgos.2021.09.007 PMC9616240

[80] Karlsson Linnér R, Biroli P, Kong E, Meddens SFW, Wedow R, Fontana MA, Genome-wide association analyses of risk tolerance and risky behaviors in over 1 million individuals identify hundreds of loci and shared genetic influences. Nature Genetics. 2019;51(2):245–57. 30643258 10.1038/s41588-018-0309-3 PMC6713272

[81] Karlsson Linnér R, Mallard TT, Barr PB, Sanchez-Roige S, Madole JW, Driver MN, Multivariate analysis of 1.5 million people identifies genetic associations with traits related to self-regulation and addiction. Nature Neuroscience. 2021;24(10):1367–76. 34446935 10.1038/s41593-021-00908-3 PMC8484054

[82] Okbay A, Wu Y, Wang N, Jayashankar H, Bennett M, Nehzati SM, Polygenic prediction of educational attainment within and between families from genome-wide association analyses in 3 million individuals. Nature Genetics. 2022;54(4):437–49. 35361970 10.1038/s41588-022-01016-z PMC9005349

[83] Watanabe K, Jansen PR, Savage JE, Nandakumar P, Wang X, Agee M, Genome-wide meta-analysis of insomnia prioritizes genes associated with metabolic and psychiatric pathways. Nature Genetics. 2022;54(8):1125–32. 35835914 10.1038/s41588-022-01124-w

[84] Blokland GAM, Grove J, Chen C-Y, Cotsapas C, Tobet S, Handa R, Sex-Dependent Shared and Nonshared Genetic Architecture Across Mood and Psychotic Disorders. Biological Psychiatry. 2022;91(1):102–17. 34099189 10.1016/j.biopsych.2021.02.972 PMC8458480

[85] Aberg KA, Liu Y, Bukszár J, McClay JL, Khachane AN, Andreassen OA, A Comprehensive Family-Based Replication Study of Schizophrenia Genes. JAMA Psychiatry. 2013;70(6):573–81. 23894747 10.1001/jamapsychiatry.2013.288 PMC5297889

[86] Jansen S, van der Werf IM, Innes AM, Afenjar A, Agrawal PB, Anderson IJ, De novo variants in FBXO11 cause a syndromic form of intellectual disability with behavioral problems and dysmorphisms. European Journal of Human Genetics. 2019;27(5):738–46. 30679813 10.1038/s41431-018-0292-2 PMC6462006

[87] Gregor A, Sadleir LG, Asadollahi R, Azzarello-Burri S, Battaglia A, Ousager LB, <em>De Novo</em> Variants in the F-Box Protein <em>FBXO11</em> in 20 Individuals with a Variable Neurodevelopmental Disorder. The American Journal of Human Genetics. 2018;103(2):305–16. 30057029 10.1016/j.ajhg.2018.07.003 PMC6080769

[88] Gouveia C, Gibbons E, Dehghani N, Eapen J, Guerreiro R, Bras J. Genome-wide association of polygenic risk extremes for Alzheimer’s disease in the UK Biobank. Scientific Reports. 2022;12(1):8404. 35589863 10.1038/s41598-022-12391-2 PMC9120074

[89] Dawson DW, Volpert OV, Gillis P, Crawford SE, Xu HJ, Benedict W, Pigment Epithelium-Derived Factor: A Potent Inhibitor of Angiogenesis. Science. 1999;285(5425):245–8. 10398599 10.1126/science.285.5425.245

[90] Tian T, Yang Y, Xu B, Qin Y, Zang G, Zhou C, Pigment epithelium-derived factor alleviates depressive-like behaviors in mice by modulating adult hippocampal synaptic growth and Wnt pathway. Progress in Neuro-Psychopharmacology and Biological Psychiatry. 2020;98:109792. 31676463 10.1016/j.pnpbp.2019.109792

[91] Bai M, Yu H, Chen C, Xu X, He Y, Wang Y, Pigment epithelium-derived factor may induce antidepressant phenotypes in mice by the prefrontal cortex. Neuroscience Letters. 2022;771:136423. 34965441 10.1016/j.neulet.2021.136423

[92] Mann JJ. Neurobiology of suicidal behaviour. Nature Reviews Neuroscience. 2003;4(10):819–28. 14523381 10.1038/nrn1220

[93] Arloth J, Eraslan G, Andlauer TFM, Martins J, Iurato S, Kühnel B, DeepWAS: Multivariate genotype-phenotype associations by directly integrating regulatory information using deep learning. PLOS Computational Biology. 2020;16(2):e1007616. 32012148 10.1371/journal.pcbi.1007616 PMC7043350

[94] Gana S, Veggiotti P, Sciacca G, Fedeli C, Bersano A, Micieli G, 19q13.11 cryptic deletion: description of two new cases and indication for a role of WTIP haploinsufficiency in hypospadias. European Journal of Human Genetics. 2012;20(8):852–6. 22378287 10.1038/ejhg.2012.19 PMC3400733

[95] Tazawa S, Yamato T, Fujikura H, Hiratochi M, Itoh F, Tomae M, SLC5A9/SGLT4, a new Na+-dependent glucose transporter, is an essential transporter for mannose, 1,5-anhydro-D-glucitol, and fructose. Life Sciences. 2005;76(9):1039–50. 15607332 10.1016/j.lfs.2004.10.016

[96] Brouwer RM, Klein M, Grasby KL, Schnack HG, Jahanshad N, Teeuw J, Genetic variants associated with longitudinal changes in brain structure across the lifespan. Nature Neuroscience. 2022;25(4):421–32. 35383335 10.1038/s41593-022-01042-4 PMC10040206

[97] Tahir UA, Katz DH, Avila-Pachecho J, Bick AG, Pampana A, Robbins JM, Whole Genome Association Study of the Plasma Metabolome Identifies Metabolites Linked to Cardiometabolic Disease in Black Individuals. Nature Communications. 2022;13(1):4923. 10.1038/s41467-022-32275-3 PMC9395431 35995766

[98] Argos M, Tong L, Pierce BL, Rakibuz-Zaman M, Ahmed A, Islam T, Genome-wide association study of smoking behaviours among Bangladeshi adults. Journal of Medical Genetics. 2014;51(5):327. 24665060 10.1136/jmedgenet-2013-102151 PMC4126189

[99] van der Meer D, Kaufmann T, Shadrin AA, Makowski C, Frei O, Roelfs D, The genetic architecture of human cortical folding. Science Advances. 2022;7(51):eabj9446. 10.1126/sciadv.abj9446 PMC8673767 34910505

[100] Greenwood TA, Lazzeroni LC, Maihofer AX, Swerdlow NR, Calkins ME, Freedman R, Genome-wide Association of Endophenotypes for Schizophrenia From the Consortium on the Genetics of Schizophrenia (COGS) Study. JAMA Psychiatry. 2019;76(12):1274–84. 31596458 10.1001/jamapsychiatry.2019.2850 PMC6802253

[101] Li QS, Parrado AR, Samtani MN, Narayan VA, Alzheimer’s Disease Neuroimaging I. Variations in the FRA10AC1 Fragile Site and 15q21 Are Associated with Cerebrospinal Fluid Aβ1–42 Level. PLOS ONE. 2015;10(8):e0134000. 26252872 10.1371/journal.pone.0134000 PMC4529186

[102] Zhang H, Kranzler HR, Yang BZ, Luo X, Gelernter J. The OPRD1 and OPRK1 loci in alcohol or drug dependence: OPRD1 variation modulates substance dependence risk. Molecular Psychiatry. 2008;13(5):531–43. 17622222 10.1038/sj.mp.4002035 PMC3163084

[103] Levran O, Londono D, O’Hara K, Nielsen DA, Peles E, Rotrosen J, Genetic susceptibility to heroin addiction: a candidate gene association study. Genes, Brain and Behavior. 2008;7(7):720–9. 18518925 10.1111/j.1601-183X.2008.00410.x PMC2885890

[104] Lam M, Chen C-Y, Li Z, Martin AR, Bryois J, Ma X, Comparative genetic architectures of schizophrenia in East Asian and European populations. Nature Genetics. 2019;51(12):1670–8. 31740837 10.1038/s41588-019-0512-x PMC6885121

[105] Kim N, Mickelson JB, Brenner BE, Haws CA, Yurgelun-Todd DA, Renshaw PF. Altitude, Gun Ownership, Rural Areas, and Suicide. American Journal of Psychiatry. 2011;168(1):49–54. 20843869 10.1176/appi.ajp.2010.10020289 PMC4643668

[106] Gao G, Li Y, Gee S, Dudley A, Fant J, Crosson C, Down-regulation of Vascular Endothelial Growth Factor and Up-regulation of Pigment Epithelium-derived Factor: A POSSIBLE MECHANISM FOR THE ANTI-ANGIOGENIC ACTIVITY OF PLASMINOGEN KRINGLE 5 *. Journal of Biological Chemistry. 2002;277(11):9492–7. 11782462 10.1074/jbc.M108004200

[107] Kang I, Kondo D, Kim J, Lyoo IK, Yurgelun-Todd D, Hwang J, Elevating the level of hypoxia inducible factor may be a new potential target for the treatment of depression. Medical Hypotheses. 2021;146:110398. 33246695 10.1016/j.mehy.2020.110398

[108] Bae W-J, Shin M-R, Kang S-K, Zhang J, Kim J-Y, Lee S-C, HIF-2 Inhibition Supresses Inflammatory Responses and Osteoclastic Differentiation in Human Periodontal Ligament Cells. Journal of Cellular Biochemistry. 2015;116(7):1241–55. 25565665 10.1002/jcb.25078

[109] Morris NL, Yeligar SM. Role of HIF-1α in Alcohol-Mediated Multiple Organ Dysfunction. Biomolecules [Internet]. 2018; 8(4). 10.3390/biom8040170 PMC6316086 30544759

[110] Krishnan HR, Zhang H, Chen Y, Bohnsack JP, Shieh AW, Kusumo H, Unraveling the epigenomic and transcriptomic interplay during alcohol-induced anxiolysis. Molecular Psychiatry. 2022;27(11):4624–32. 36089615 10.1038/s41380-022-01732-2 PMC9734037

[111] Caldwell HK. Neurobiology of Sociability. In: López-Larrea C, editor. Sensing in Nature. New York, NY: Springer US; 2012. p. 187–205. 10.1007/978-1-4614-1704-0_12 PMC4146394 22399403

[112] Kelly AM, Goodson JL. Social functions of individual vasopressin–oxytocin cell groups in vertebrates: What do we really know? Frontiers in Neuroendocrinology. 2014;35(4):512–29. 24813923 10.1016/j.yfrne.2014.04.005

[113] Oquendo MA, Sullivan GM, Sudol K, Baca-Garcia E, Stanley BH, Sublette ME, Toward a biosignature for suicide. Am J Psychiatry. 2014;171(12):1259–77. 25263730 10.1176/appi.ajp.2014.14020194 PMC4356635

[114] Mann JJ, Arango VA, Avenevoli S, Brent DA, Champagne FA, Clayton P, Candidate Endophenotypes for Genetic Studies of Suicidal Behavior. Biological Psychiatry. 2009;65(7):556–63. 19201395 10.1016/j.biopsych.2008.11.021 PMC3271953

[115] Gibbons JL. Electrolytes and Depressive Illness. Postgraduate Medical Journal. 1963;39(447):19. 13947657 10.1136/pgmj.39.447.19 PMC2482182

[116] Ellis GG, Coppen A, Glen AIM. Urine concentration in depressive illness. Journal of Neurology, Neurosurgery &amp; Psychiatry. 1971;34(1):30. 5551692 10.1136/jnnp.34.1.30 PMC493683

[117] Harper KM, Knapp DJ, Criswell HE, Breese GR. Vasopressin and alcohol: a multifaceted relationship. Psychopharmacology. 2018;235(12):3363–79. 30392132 10.1007/s00213-018-5099-x PMC6286152

[118] Zhao F-Q, Keating FA. Functional Properties and Genomics of Glucose Transporters. Current Genomics. 2007;8(2):113–28. 18660845 10.2174/138920207780368187 PMC2435356

[119] Koponen H, Kautiainen H, Leppänen E, Mäntyselkä P, Vanhala M. Association between suicidal behaviour and impaired glucose metabolism in depressive disorders. BMC Psychiatry. 2015;15(1):163. 26199013 10.1186/s12888-015-0567-x PMC4509469

[120] Dong R, Haque A, Wu HE, Placide J, Yu L, Zhang X. Sex differences in the association between suicide attempts and glucose disturbances in first-episode and drug naive patients with major depressive disorder. Journal of Affective Disorders. 2021;292:559–64. 34147968 10.1016/j.jad.2021.05.110

[121] Gilder DA, Wall TL, Ehlers CL. Comorbidity of select anxiety and affective disorders with alcohol dependence in southwest California Indians. Alcohol Clin Exp Res. 2004;28(12):1805–13. 15608596 10.1097/01.alc.0000148116.27875.b0

